# Analysis of secondhand smoke exposure and harm awareness among non-smoking individuals aged 15–44 years in Jilin Province: A cross-sectional study

**DOI:** 10.18332/tid/208809

**Published:** 2025-10-07

**Authors:** Wenling Li, Jianying Jiang, Ruolin Li, Ling Zhang, Bing Jia, Qiao Zhang, Xiaobo Qian

**Affiliations:** 1 Public Health Department, Jilin Qianwei Hospital, Changchun, China; 2 Changchun Medical College, Changchun, China; 3 Project Management Office, Institute of Health Education of Jilin Province, Changchun, China; 4 Health Education Division, Jilin City Center for Disease Control and Prevention, Jilin, China

**Keywords:** secondhand smoke exposure, non-smokers, cross-sectional study, China

## Abstract

**INTRODUCTION:**

In recent years, alongside the Healthy China Initiative, extensive nationwide efforts have been undertaken to enhance the health literacy of the population. Health knowledge dissemination has emerged as a key approach within these efforts. This study focused on non-smokers aged 15–44 years in Jilin Province, China. It analyzed their patterns of secondhand smoke (SHS) exposure and their level of awareness regarding its risks.

**METHODS:**

The study selected non-smoking permanent residents aged 15–44 years from the 2020 Jilin Province Adult Tobacco Survey database as its subjects. Following the requirements of the China Adult Tobacco Survey Protocol, the project employed a multistage stratified cluster random sampling method. This involved: selecting 10 surveillance sites from all of the province's districts or counties; choosing 3 sub-districts/townships within each surveillance site; selecting 2 neighborhood or village committees within each sub-district or township; and randomly sampling 120 households from each neighborhood or village committee. This resulted in a total sample of 7200 households province-wide. One resident aged ≥15 years was randomly selected from each household for a face-to-face in-home interview. The present analysis utilized eligible questionnaires from individuals within the target age group 15–44 years.

**RESULTS:**

The prevalence of secondhand smoke (SHS) exposure among surveyed non-smoking residents in Jilin Province was 59.16% (95% CI: 52.87–65.17). Awareness rates of specific SHS health risks were as follows: 76.16% (95% CI: 59.77–87.30) knew SHS causes lung cancer in adults; 61.95% (95% CI: 52.26–70.77) knew SHS causes lung diseases in children; and 49.21% (95% CI: 34.99– 63.56) knew SHS causes heart disease in adults. However, only 42.26% (95% CI: 32.60–52.56) were aware that SHS causes all three conditions (heart disease in adults, lung diseases in children, and lung cancer in adults). SHS exposure rates varied significantly by location: restaurants had the highest exposure rate (50.85%) , homes (37.72%), and public transport (5.01%).

**CONCLUSIONS:**

SHS exposure prevalence among residents aged 15–44 years in Jilin Province remained high, while comprehensive awareness of its associated health risks was relatively low. A discrepancy existed between possessing knowledge about SHS harms and translating that knowledge into protective behaviors or reduced exposure. Intervention efforts should focus on priority venues, intensify the dissemination of core knowledge on tobacco hazards, implement targeted health promotion activities for key populations, and foster supportive smoke-free environments.

## INTRODUCTION

Secondhand smoke (SHS) exposure is globally recognized as one of the major and preventable health threats. According to the World Health Organization (WHO) data, tobacco causes over 8 million deaths worldwide annually, including an estimated 1.3 million non-smokers exposed to SHS^1^. Tobacco contains >7000 chemicals, with at least 70 identified carcinogens such as benzene, formaldehyde, and nitrosamines^2,3^. SHS similarly contains numerous harmful compounds including benzene, toluene, butane, cadmium, and cyanide^4^. Scientific evidence confirms that exposure to tobacco smoke causes death, disease, and disability in non-smokers. Long-term exposure significantly increases risks of lung cancer, cardiovascular diseases, chronic obstructive pulmonary disease (COPD), and respiratory infections in children^5-9^. There is no safe level of exposure secondhand smoke^10^. Neonates exposed prenatally or postnatally face elevated risks of preterm birth, low birth weight, and sudden infant death syndrome^11,12^. Despite smoke-free policies in public spaces across many countries, SHS exposure remains persistently high among non-smokers, particularly in low- and middle-income countries where household and workplace exposure are most severe^13^.

The 15–44 years age cohort encompasses adolescents, reproductive-age women, and working adults – demographic groups whose health status critically influences workforce productivity, reproductive health security, and chronic disease prevention. Regarding secondhand smoke (SHS) exposure, this population faces a triple-risk burden across domestic, occupational, and social environments^14,15^. In recent years, The Healthy China Initiative has prioritized nationwide health literacy campaigns, with health knowledge dissemination as a key strategy^16,17^. This study examined non-smokers aged 15–44 years in Jilin Province, China, to analyze SHS exposure patterns and risk awareness levels; investigate relationships between exposure to health science information, knowledge of SHS harms, and actual SHS exposure and to provide evidence to inform local policymakers and enhance tobacco control strategies.

## METHODS

### Data sources

This study selected 1567 permanent non-smoking residents aged 15–44 years from the 2020 Jilin Province Adult Tobacco Survey (JPATS) database as the study population. The JPATS was an integral component of the China Adult Tobacco Survey (CATS). The CATS was a systematic national public health surveillance program designed to collect data on adult tobacco use. Its objectives were to monitor tobacco use trends, evaluate the effectiveness of tobacco control policies, and provide critical evidence for formulating new public health strategies. The JPATS dataset provided province-level representative surveillance data for Jilin. For this analysis, individuals residing in city districts were classified as the urban population, while those living in counties or county-level cities were classified as the rural population.

### Sampling

The study selected non-smoking permanent residents aged 15–44 years from the 2020 Jilin Province Adult Tobacco Survey database as its subjects. Following the requirements of the China Adult Tobacco Survey Protocol^18^, the project employed a multistage stratified cluster random sampling method. This involved: selecting 10 surveillance sites from all of the province’s districts or counties; choosing 3 sub-districts/townships within each surveillance site; selecting 2 neighborhood or village committees within each sub-district or township; and randomly sampling 120 households from each neighborhood or village committee. This resulted in a total sample of 7200 households province-wide. One resident aged ≥15 years was randomly selected from each household for a face-to-face in-home interview. The present analysis utilized eligible questionnaires from individuals within the target age group 15–44 years.

### Survey methods

The survey employed in-person household interviews conducted by field investigators using handheld computers (IPAQ). Data collection utilized the standard questionnaire from the Global Tobacco Surveillance System (GTSS) – specifically, the China Adult Tobacco Survey Questionnaire (TQS).

### Survey contents

The questionnaire covered: basic demographic characteristics, smoking behavior, e-cigarette use, smokeless tobacco products, cessation attempts, secondhand smoke (SHS) exposure, cigarette economics, and media exposure. This study specifically focused on sections pertaining to SHS exposure, media influence, and knowledge, attitudes, and perceptions regarding tobacco.

### Definitions

*Population SHS exposure*

Defined as non-smokers exposed to smoke emanating from the burning end of a cigarette or exhaled by smokers on at least one day per week^19^. SHS exposure rate = (number of non-smokers exposed to SHS/total number of non-smokers)×100%.

*Location-specific SHS exposure*

Observing someone smoke within the past 30 days in indoor public places, workplaces, public transport vehicles, or their own home.

### Statistical analysis

Sample weights were calculated according to the sampling procedures. All statistical analyses were performed using the complex sampling module in SPSS22.0, accounting for these weights. Sample characteristics were described using frequencies and percentages for categorical variables and means with standard deviations (SD) for continuous variables. Chi-squared tests (χ^2^) were employed to examine associations between secondhand smoke (SHS) exposure rates, awareness of SHS harms, and sociodemographic characteristics. Adjusted odds ratios with corresponding 95% confidence intervals (95% CI) were calculated. Statistical significance was defined as a two-tailed p<0.05.

## RESULTS

### General situation

The study included 1567 participants. After applying sampling weights, the data represented a population of 8204485 individuals within the target age group. The mean age was 34.44 years (SD=6.77). The study population comprised 595 males (37.97%) and 972 females (62.03%). Age distribution analysis revealed: 129 participants (8.23%) aged 15–24 years, 202 (12.89%) 25–29 years, 439 (28.02%) 30–34 years, 357 (22.78%) 35–39 years, and 440 (28.08%) 40–44 years. Education level included: 111 individuals (7.08%) with primary school education or lower, 473 (30.19%) with junior high school education, 350 (22.34%) high school graduates, and 633 (40.40%) holding college degrees or higher qualifications. Residence distribution showed 1193 participants (76.13%) from urban areas and 374 (23.87%) from rural settings. Occupational categories were represented as follows: 297 individuals (18.95%) employed in agriculture, forestry, animal husbandry, fishery, or water conservancy; 195 (12.44%) working as government/institutional staff, teachers, or medical personnel; 397 (25.34%) engaged in enterprise, business, or service industries; 244 (15.57%) comprising students, military personnel, or unemployed/inactive individuals; and 399 (25.46%) belonging to other occupational groups.

Occupational data were not reported for 35 participants.

### Weekly SHS exposure among non-smokers by characteristics

The prevalence of SHS exposure among surveyed non-smokers in Jilin Province was 59.16% (95% CI: 52.87–65.17) (Table 1). A statistically significant difference (p<0.05) in SHS exposure was observed between urban and rural residents. Rural residents had a significantly higher SHS exposure rate compared to urban residents (χ^2^=6.303, p =0.036).

**Table 1:** Comparison of weekly secondhand smoke (SHS) exposure patterns among non-smoking residents by sociodemographic characteristics, Jilin Province, China (2020)

*Characteristics*	*SHS exposure* *Unweighted n, % (95% CI)*						*Total* *n*	*Exposure* *% (95% CI)*	*χ^2^*	*p*
	*Almost daily*		*4–6 days/week*		*1–3 days/week*					
**Sex**									0.485	0.752
Male	104	19.14 (11.73–29.68)	55	8.89 (4.19–17.89)	150	25.21 (17.57–34.77)	309	53.24 (39.45–66.56)		
Female	222	29.76 (18.33–44.44)	75	8.93 (5.39–14.43)	196	23.91 (10.73–45.09)	493	62.60 (53.73–70.69)		
**Age** (years)									0.351	0.738
15–24	29	27.04 (9.78–55.87)	7	5.70 (1.30–21.69)	30	28.57 (13.56–50.48)	66	61.30 (45.59–74.97)		
25–29	43	25.75 (12.12–46.59)	21	13.31 (4.44–33.66)	45	23.57 (10.46–44.87)	109	62.63 (46.94–76.05)		
30–34	89	25.05 (12.89–43.03)	37	10.33 (6.19–16.76)	87	17.96 (11.72–26.55)	213	53.35 (39.07–67.10)		
35–39	75	24.35 (19.36–30.14)	33	7.80 (3.95–14.81)	85	27.67 (15.8–43.81)	193	59.82 (47.00–71.43)		
40–44	90	25.72 (17.70–35.80)	32	9.70 (4.84–18.50)	99	23.25 (14.97–34.27)	221	58.67 (48.49–68.17)		
**Education level**									0.892	0.419
Primary school and lower	32	36.19 (25.71–48.17)	9	7.66 (3.93–14.39)	22	19.8 (12.24–30.43)	63	63.65 (57.20–69.64)		
Junior high school	119	32.65 (19.29–49.59)	45	9.10 (4.86~16.39)	94	22.2 (13.06~35.14)	258	63.95 (49.96~75.92)		
High school	57	15.48 (4.69–40.57)	29	12.72 (5.63–26.26)	81	26.28 (17.77–37.04)	167	54.49 (43.83–64.76)		
College or higher	118	17.33 (10.52–27.22)	47	5.92 (4.22–8.23)	149	28.75 (11.99–54.44)	314	52.00 (31.66–71.70)		
**Residence**									6.303	0.036*
Urban	226	21.87 (14.32–31.91)	82	6.79 (4.22–10.76)	252	18.77 (10.43–31.44)	560	47.43 (33.59–61.67)		
Rural	100	28.01 (15.32–45.55)	48	10.23 (5.72–17.63)	94	27.90 (14.88–46.13)	242	66.13 (59.62–72.08)		
**Exposed to tobacco control (TC) campaigns**									0.193	0.672
No	129	22.45(10.99–40.42)	58	8.06 (4.08–15.32)	134	30.50 (15.55–51.12)	321	61.01 (51.43–69.82)		
Yes	197	28.92 (19.63–40.41)	72	9.78 (6.14–15.22)	212	18.22 (13.18–24.64)	481	56.92 (41.52–71.09)		
**Occupation^a^**									1.411	0.273
Agriculture/forestry/fishery workers	74	25.77 (14.30–41.93)	39	10.62 (5.00–21.15)	74	29.05 (13.03–52.81)	187	65.44 (52.56–76.39)		
Government/institution staff/teachers/medical personnel	36	14.76 (9.00–23.27)	8	2.63 (1.03–6.57)	58	31.87 (16.65–52.28)	102	49.26 (28.54–70.24)		
Enterprise/business/service workers	115	39.81 (27.87–53.11)	38	10.35 (3.81–25.17)	83	15.91 (10.62–23.13)	236	66.07 (51.45–78.15)		
Students/military/unemployed	38	14.70 (8.18–25.00)	15	8.23 (2.35–25.03)	41	24.62 (16.11–35.71)	94	47.54 (32.94–62.58)		
Other	61	32.10 (12.55–60.90)	29	6.90 (3.39–13.54)	84	15.44 (6.17–33.67)	174	54.45 (31.66–75.51)		
**Total**	326	25.66 (15.93–38.60)	130	8.91 (5.48–14.17)	346	24.41 (15.49–36.27)	802	59.16 (52.87–65.17)		

a Some data missing.

* p<0.05.

### Awareness of SHS health harms among non-smokers

Awareness of SHS health harms is shown in Table 2. About 76.16% (95% CI: 59.77–87.30) knew SHS causes lung cancer in adults; 61.95% (95% CI: 52.26–70.77) knew SHS causes childhood lung diseases; 49.21% (95% CI: 34.99–63.56) knew SHS causes heart disease in adults; However, only 42.26% (95% CI: 32.60–52.56) were aware that SHS causes all three conditions (heart disease in adults, childhood lung diseases, and lung cancer in adults.

**Table 2:** Awareness of SHS health harms among non-smoking residents, Jilin Province, China (2020)

*Variables*	*Heart disease in* *adults*	*Childhood lung* *diseases*	*Lung cancer in adults*	*All three conditions* *combined*
	*Awareness % (95% CI)*			
**Sex**				
Male	46.23 (28.71–64.73)	59.83 (49.32–69.51)	71.92 (53.23–85.21)	40.56 (27.13–55.56)
Female	51.54 (38.85–64.03)	63.22 (51.65–73.44)	78.90 (64.08–88.69)	43.34 (35.38–51.65)
χ^2^	1.398	0.450	11.185	0.425
p	0.271	0.521	0.010*	0.533
**Age** (years)				
15–24	60.57 (36.69–80.28)	67.88 (45.95–84.01)	81.20 (67.77–89.87)	55.66 (31.40–77.49)
25–29	42.32 (33.83–51.29)	60.27 (47.78–71.55)	76.86 (53.36–90.61)	36.12 (27.18–46.14)
30–34	55.22 (34.16–74.56)	61.79 (51.57–71.07)	80.02 (57.96–92.09)	45.25 (29.26–62.28)
35–39	47.68 (36.45–59.14)	60.87 (47.82–72.53)	74.39 (51.24–88.92)	36.41 (30.75–42.48)
40–44	38.74 (29.14–49.30)	57.62 (46.13–68.34)	69.24 (51.85–82.48)	33.98 (25.05–44.20)
χ^2^	4.560	0.623	1.436	2.458
p	0.019*	0.536	0.266	0.130
**Education level**				
Primary school and lower	29.26 (16.26–46.83)	36.67 (19.87–57.49)	46.68 (32.66–61.24)	25.87 (14.03–42.72)
Junior high school	42.92 (32.12–54.44)	51.90 (46.40–57.35)	71.51 (52.75–84.94)	30.91 (21.03–42.91)
High school	50.77 (30.53–70.76)	70.12 (56.79–80.73)	86.77 (71.57–94.47)	46.06 (28.84–64.28)
College or higher	68.95 (53.41–81.14)	83.78 (74.33–90.21)	88.70 (82.12–93.06)	66.27 (51.22–78.62)
χ^2^	9.711	16.673	13.382	8.917
p	0.001**	<0.001***	0.001**	0.001**
**Residence**				
Urban	64.20 (51.12–75.47)	77.89 (72.07–82.78)	84.73 (78.43–89.44)	60.79 (48.99–71.45)
Rural	40.38 (24.29–58.84)	52.02 (38.2–65.54)	70.93 (48.11–86.53)	30.80 (23.31–39.46)
χ^2^	9.544	20.435	4.391	45.948
p	0.015*	0.002**	0.069	<0.001***
**SHS exposure**				
No	53.23 (36.25–69.50)	60.03 (54.23–65.56)	77.49 (60.63–88.50)	43.55 (34.84–52.68)
Yes	46.88 (33.46–60.76)	63.22 (46.7–77.13)	75.31 (58.49–86.85)	41.37 (30.09–53.64)
χ^2^	2.606	0.175	0.429	0.376
p	0.145	0.687	0.531	0.557
**Exposed to tobacco control (TC) campaigns**				
No	34.63 (21.85–50.09)	51.43 (33.57–68.93)	69.42 (48.77–84.41)	30.52 (22.35–40.14)
Yes	64.58 (46.79–79.08)	72.56 (64.77–79.18)	83.1 (67.82–91.98)	54.19 (39.96–67.77)
χ^2^	22.558	5.384	9.678	14.618
p	0.001**	0.049*	0.014**	0.005**
**Occupation**				
Agriculture/forestry/fishery workers	38.79 (21.14–59.97)	47.15 (35.98–58.61)	66.41 (40.14–85.36)	26.57 (19.26–35.44)
Government/institution staff/teachers/medical personnel	68.3 (46.04–84.48)	93.34 (87.38–96.6)	94.60 (90.65–96.94)	66.64 (44.19–83.44)
Enterprise/business/service workers	65.25 (52.92–75.83)	79.32 (70.88–85.8)	87.64 (76.86–93.80)	58.65 (49.12–67.57)
Students/military/unemployed	58.65 (36.24–77.97)	76.19 (58.86–87.74)	84.11 (71.56–91.75)	58.02 (35.84–77.38)
Other	42.63 (24.79–62.62)	54.52 (29.97–77.06)	74.06 (59.78–84.58)	39.52 (22.88–59.01)
χ^2^	3.158	9.434	5.610	7.517
p	0.061	0.003**	0.020*	0.006**
**Total**	49.21 (34.99–63.56)	61.95 (52.26–70.77)	76.16 (59.77–87.30)	42.26 (32.60–52.56)

* p<0.05.

** p<0.01.

*** p<0.001.

### SHS exposure across different venues

**Figure 1:**
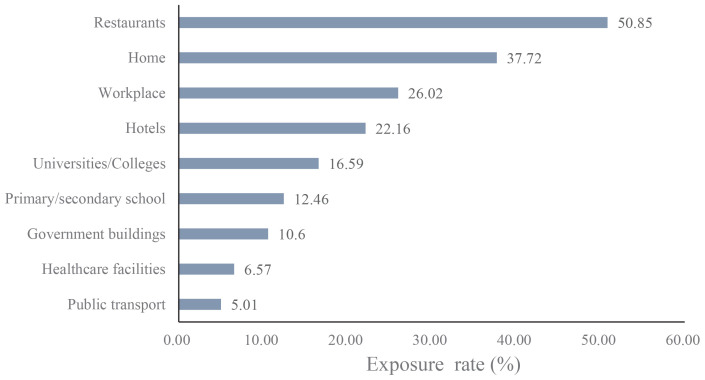
Exposure in different venues

SHS exposure by location is shown in Table 3 and Figure 1. The results revealed significant variation: restaurants exhibited the highest exposure rate at 50.85%; homes showed the second-highest exposure (37.72%); and public transport had the lowest exposure rate (5.01%).

**Table 3:** SHS exposure in different venues among non-smokers, Jilin Province, China (2020)

*Venue*	*Total* *n*	*Exposed* *n*	*Exposure rate* *% (95% CI)*
Home	1527	401	37.72 (25.21–52.12)
Workplace	855	211	26.02 (17.87–36.26)
Government buildings	180	26	10.60 (4.58–22.65)
Healthcare facilities	271	27	6.57 (3.19–13.05)
Restaurants	791	471	50.85 (37.86–63.74)
Public transport	574	32	5.01 (2.42–10.08)
Universities/colleges	50	7	16.59 (4.48–45.76)
Primary/secondary school	284	45	12.46 (5.88–24.50)
Hotels	43	12	22.16 (12.08–37.10)

### Household SHS exposure among females

Household SHS exposure rates are shown in Table 4. Those with college degrees or higher demonstrated significantly lower exposure. Urban–rural disparities were pronounced: females in urban areas had substantially lower household exposure than their rural counterparts.

**Table 4:** Household secondhand smoke exposure among non-smoking females, Jilin Province, China (2020) (N=945)

*Variables*	*Exposed* *n*	*Exposure rate* *% (95% CI)*	*χ^2^*	*p*
**Age** (years)			0.185	0.898
15–24	18	38.01 (16.00–66.38)		
25–29	38	45.44 (29.04–62.89)		
30–34	75	45.05 (27.27–64.19)		
35–39	74	40.63 (21.60–62.96)		
40–44	69	38.12 (24.12–54.41)		
**Education level**			7.315	0.008**
Primary school and lower	30	44.58 (27.71–62.79)		
Junior high school	113	49.94 (31.20–68.70)		
High school	52	50.40 (34.04–66.67)		
College or higher	79	17.87 (9.42–31.27)		
**Residence**			9.325	0.016*
Urban	161	26.14 (17.86–36.55)		
Rural	113	49.51 (33.31–65.82)		
**Exposed to tobacco control (TC) campaigns**			2.310	0.167
No	107	35.22 (22.24–50.83)		
Yes	167	47.01 (33.25–61.24)		
**Occupation**			0.850	0.457
Agriculture/forestry/fishery workers	83	44.66 (21.38–70.55)		
Government/institution staff/teachers/medical personnel	30	20.59 (9.23–39.79)		
Enterprise/business/service workers	70	46.51 (35.77–57.59)		
Students/military/unemployed	43	41.88 (28.59–56.46)		
Other	47	36.37 (15.39–64.23)		
Total	274	40.98 (29.19–53.91)		

* p<0.05.

** p<0.01.

## DISCUSSION

The study revealed a 59.16% secondhand smoke (SHS) exposure prevalence among non-smokers aged 15–44 years in Jilin Province, lower than the 2016–2017 rates (15–24 years: 61.4%; 25–44 years: 67.8%)^20^.This reduction may be attributed to enhanced tobacco control initiatives, including public health education and tobacco tax increases, and smoking bans in public spaces^21,22^. Notably, rural residents demonstrated significantly higher SHS exposure than their urban counterparts, potentially reflecting greater tobacco accessibility, lifestyle patterns, and limited SHS risk awareness^23,24^.

Despite robust evidence linking SHS to multiple pathologies^4-9^. Population awareness of SHS-induced adult cardiovascular disease remained suboptimal – consistent with prior reports^25^. Females exhibited higher recognition than males regarding SHS-related lung cancer, possibly attributable to greater health information engagement and family health prioritization^26^.

Adolescents and young adults (15–24 years) demonstrated significantly higher awareness that secondhand smoke (SHS) caused cardiovascular disease in adults. This likely stemmed from their greater accessibility to health education channels such as school-based programs and social media platforms, combined with heightened receptiveness to emerging health knowledge^27,28^. A positive educational gradient was observed in SHS risk awareness, where higher academic attainment correlated with enhanced hazard recognition. This pattern underscored education’s critical role in advancing public health literacy, as university-educated individuals typically exhibit superior capacity to acquire and interpret complex health information.

Urban residents consistently outperformed their rural counterparts in recognizing SHS links to diseases. This disparity reflected urban advantages in educational infrastructure, healthcare accessibility, and information dissemination networks.

Exposure to tobacco-related health communication effectively elevated public risk awareness, demonstrating targeted education’s efficacy. Similarly, professional and technical personnel showed heightened recognition of SHS-induced pediatric lung diseases, adult lung cancer, and combined health risks – attributable to their advanced academic backgrounds and occupational requirements for maintaining current health knowledge.

Critically, however, no statistically significant association existed between exposure to health education and actual SHS avoidance behaviors. This fundamental knowledge–behavior gap highlighted the persistent disconnection between risk awareness and protective action implementation among the population.

Restaurants demonstrated the highest prevalence of secondhand smoke (SHS) exposure at 50.85%, followed by households (37.72%), with public transportation exhibiting the lowest exposure rate at 5.01%. This distribution pattern reflects distinct environmental determinants across venues. In Northeast China’s prolonged and harsh winters, restaurants become critical social hubs where inadequate enforcement of smoke-free policies facilitates elevated smoking prevalence during indoor congregation for dining and socialization^29,30^. The substantial household exposure rate signals insufficient recognition of SHS hazards within domestic settings and limited cessation support systems. Conversely, the minimal exposure observed in public transport systems stems from stringent smoke-free regulations effectively implemented in urban centers, compounded by heightened public scrutiny in these confined spaces.

College-educated females exhibited significantly lower rates of secondhand smoke (SHS) exposure. This phenomenon was likely attributable to their heightened sensitivity to health information, which enabled more effective acquisition, comprehension, and application of knowledge regarding SHS risks^31^. This demographic demonstrates greater propensity to adopt protective measures against SHS exposure. Furthermore, their superior economic means and social resources facilitated the establishment of smoke-free domestic environments. These findings underscore the critical importance of advancing educational attainment, particularly among women, as a strategic intervention for SHS reduction^18^. Education transcends mere knowledge dissemination by serving as a catalyst for adopting healthier lifestyles. Consequently, targeted health education initiatives for less-educated populations represent an essential public health priority.

Urban females experience substantially lower SHS exposure compared to their rural counterparts. Urban advantages included more stringent tobacco control regulations – encompassing comprehensive public place smoking bans – coupled with robust health education infrastructure featuring regular health campaigns and lectures. Enhanced accessibility to healthcare services further strengthens risk awareness and preventive practices among urban residents^32^. Conversely, rural areas confront multifaceted challenges: inadequate health education resources, limited medical facilities, and cultural norms that tolerate smoking. Higher regional tobacco use prevalence compounds these issues, while insufficient policy enforcement fails to address household SHS exposure. Bridging this inequity necessitates context-specific tobacco control strategies tailored to rural realities, supported by substantially increased governmental and organizational investments. These coordinated efforts must ensure all populations benefit equally from health-protective environments.

Notably, this study revealed no statistically significant association between exposure to tobacco control campaigns and avoidance of secondhand smoke (SHS) exposure, nor awareness of SHS health hazards and actual exposure reduction. This demonstrates a fundamental dissociation between risk perception and protective behaviors among non-smokers. The observed knowledge-behavior gap may be attributable to three interconnected factors^33-35^: implicit cultural tolerance of domestic smoking behaviors within social norms; inconsistent enforcement of smoke-free policies in public spaces; individual optimistic bias regarding perceived lowdose exposure risks.

### Limitations

This study has several limitations. First, the sample was derived from Jilin Province’s adult tobacco surveillance data. Due to factors including population aging and seasonal survey variations, the sample size was relatively small. As a population-based crosssectional survey relying on self-reported data, it lacked biomarker validation to objectively quantify exposure levels, potentially underestimating true SHS risks. Second, the observational design cannot establish causality and may suffer from residual confounding by unmeasured factors. Third, findings may have limited generalizability to other countries with differing socioeconomic contexts, tobacco control policies, and cultural norms. Finally, while key variables were considered, the analysis did not fully adjust for all potential confounders in comprehensive multivariate models – particularly nuanced urban–rural differences beyond basic residence classification. These constraints should be considered when interpreting the results.

## CONCLUSIONS

Secondhand smoke (SHS) exposure remained high among residents aged 15–44 years in Jilin Province, while awareness of its health hazards was comparatively low. Furthermore, a gap persisted between information awareness, knowledge acquisition, and behavioral change. To address this, targeted interventions should focus on high-risk settings by intensifying science communication campaigns that disseminate essential facts about tobacco risks. Health promotion initiatives for priority populations should be strengthened alongside creating supportive smoke-free environments.

## Data Availability

The data supporting this research are available from the authors on reasonable request.
